# Supplementary material for: Exocrine Pancreatic Dysfunction in Chronic Pancreatitis: A Cross-Sectional Analysis of the Prospective Evaluation of Chronic Pancreatitis for Epidemiologic and Translational Studies Study (PROCEED)
Source: Gastro Hep Adv. 2026 Sep 24;5(12):101121. doi: 10.1016/j.gastha.2026.101121 (PMC13757507; doi:10.1016/j.gastha.2026.101121)

Title: Exocrine Pancreatic Dysfunction in Chronic Pancreatitis: Analysis of the PROCEED Study

SUPPLEMENTARY CONTENT

Supplementary Table 1: Baseline characteristics of CP participants with and without EPD in whom EPD status was assessed prior to or at enrollment, separated according to FE-1 result (<100, 100-200, and ≥200mcg/g) with overall 3-group comparison (Assessed Cohort, n=419).

	**FE-1 <100 (n=260)**	**FE-1 100-200 (n=41)**	**FE-1 ≥200 (n=118)**	**P-value (100-200 vs ≥200)**	**P-value (overall, 3-group)**
**Demographics**					
Median age at enrollment, y; median (IQR)	56 (46-63)	55 (47-61)	57 (45-65)	0.48	0.74
Female sex, n (%)	114 (43.8)	20 (48.8)	63 (53.4)	0.72	0.22
White race, n (%)	215 (82.7)	39 (95.1)	101 (85.6)	0.16	0.1
BMI, kg/m2; median (IQR)	23.9 (21.0-28.0)	25.4 (21.6-28.3)	25.2 (21.8-29.3)	0.75	0.09
**Social Determinants of Health**					
Highest level of education, n (%)				0.36	0.46
HS/GED or less	90 (34.9)	13 (32.5)	34 (29.3)		
Some training past HS	88 (34.1)	16 (40.0)	36 (31.0)		
Bachelor's or graduate school	80 (31.0)	11 (27.5)	46 (39.7)		
Family income, n (%)				0.91	0.02
Less than 50k	119 (49.8)	11 (31.4)	39 (35.5)		
50-100k	67 (28.0)	11 (31.4)	31 (28.2)		
100k or greater	53 (22.2)	13 (37.1)	40 (36.4)		
Marital status, n (%)				0.29	0.26
Single	59 (22.8)	8 (19.5)	24 (20.3)		
Married	140 (54.1)	29 (70.7)	70 (59.3)		
Divorced/Separated/Widowed	60 (23.2)	4 (9.8)	24 (20.3)		
Employment status, n (%)				0.51	**0.04**
Unemployed	97 (37.9)	12 (30.8)	25 (21.6)		
Employed	95 (37.1)	17 (43.6)	55 (47.4)		
Retired	64 (25.0)	10 (25.6)	36 (31.0)		
Disability or medical leave, n (%)	89 (35.6)	10 (27.0)	23 (22.3)	0.65	**0.04**
**Clinical Characteristics**					
Smoking status, n (%)				0.46	0.07
Current	98 (37.8)	11 (26.8)	30 (25.6)		
Past	78 (30.1)	16 (39.0)	35 (29.9)		
Never	83 (32.0)	14 (34.1)	52 (44.4)		
Charlson Comorbidity Index category, n (%)				0.35	**< .01**
1	159 (61.2)	37 (90.2)	95 (80.5)		
2	79 (30.4)	2 (4.9)	16 (13.6)		
3	22 (8.5)	2 (4.9)	7 (5.9)		
Diabetes, n (%)	138 (55.0)	12 (33.3)	29 (26.1)	0.4	**< .01**
PERT usage, n (%)	184 (71.0)	16 (40.0)	29 (25.0)	0.1	**< .01**
Prior AP, n (%)	206 (82.7)	34 (85.0)	100 (85.5)	1	0.81
Duration of AP prior to enrollment, y; median (IQR)	5 (2-11)	3 (1-10)	3 (1-11)	0.95	0.06

Etiology, n (%)				0.12	**< .01**
Alcohol	126 (48.5)	14 (34.1)	25 (21.2)		
Idiopathic	92 (35.4)	14 (34.1)	61 (51.7)		
All others combined	42 (16.2)	13 (31.7)	32 (27.1)		
Pancreatic calcifications, n (%)	221 (85.0)	23 (56.1)	70 (59.3)	0.72	**< .01**
Pancreas size on imaging, n (%)				**< .01**	**< .01**
<7mm	52 (20.0)	3 (7.3)	7 (6.0)		
7-14mm	117 (45.0)	22 (53.7)	28 (23.9)		
>14mm	91 (35.0)	16 (39.0)	82 (70.1)		
Endoscopic therapy, n (%)	145 (56.4)	24 (60.0)	47 (40.5)	**0.04**	**0.01**
Pancreatic surgery, n (%)	35 (13.5)	4 (9.8)	4 (3.4)	0.21	**< .01**

Supplemental Table 2: Comparison of chronic pancreatitis-related features and outcomes between subjects with and without exocrine pancreatic dysfunction (EPD) diagnosed according to clinical features or study fecal elastase (FE-1) testing (Assessed Cohort). *Data presented as n (%) unless otherwise noted.*

	**FE-1 < 100 mcg/g (n = 260)**	**FE-1 ≥ 100 mcg/g (n = 159)**	***P*-value**	**FE-1 < 200 mcg/g (n = 301)**	**FE-1 ≥ 200 mcg/g (n = 118)**	***P*-value**
**Metabolic Bone Disease**						
Low vitamin D, *n (%)*	61 (39.9)[104]	24 (33.8)[87]	.46	69 (40.1)[129]	24 (33.8)[88]	.39
DXA result, *n (%)*						
Normal	67 (42.1)[101]	38 (47.5)[79]		78 (43.8)[123]	27 (44.3)[57]	
Osteopenia	59 (37.1)	33 (41.3)		64 (36.0)	28 (25.9)	
Osteoporosis	33 (20.8)	9 (11.2)	.19	36 (20.2)	6 (9.8)	.14
DXA result, *n (%)*						
Normal	67 (42.1)[101]	38 (47.5)[79]		78 (43.8)[123]	27 (44.3)[57]	
Abnormal	92 (57.9)	42 (52.5)	.81	100 (56.2)	34 (55.7)	1.00
Spontaneous fracture, *n (%)*	4 (1.5)[1]	4 (2.5)	.49	4 (1.3)[1]	4 (3.4)	.23
Traumatic fracture, *n (%)*						
Yes	81 (31.5)[3]	54 (34.2)[1]		96 (32.2)[3]	39 (33.3)[1]	
No	176 (68.5)	104 (65.8)	.59	202 (67.8)	78 (66.7)	.81
Traumatic fracture^ *n (%)*						
Minor	34 (33.0)[7]	27 (42.9)[1]		42 (35.3)[7]	19 (40.4)[1]	
Moderate	45 (43.7)	26 (41.3)		50 (42.0)	21 (44.7)	
Major	24 (23.3)	10 (15.9)	.35	27 (22.7)	7 (14.9)	.54
Traumatic fracture with osteopathy, *n (%)*						
Minor	15 (39.5)[3]	11 (44.0)[1]		17 (39.5)[3]	9 (45.0)[1]	
Moderate	15 (39.5)	12 (48.0)		18 (41.9)	9 (45.0)	
Major	8 (21.0)	2 (8.0)	.45	8 (18.6)	2 (10.0)	.77

^among 135 subjects with any traumatic fracture.

Note: Categorical variables are summarized as count (percentage of available data).

Note: The number of subjects with missing data is annotated in superscript.

Note: P-value is from comparison between EPD and no-EPD group, with the Fisher exact test for categorical variables.

Note: Abnormal Osteopathy includes subjects with any osteopenia or osteoporosis.

Supplementary Figure 1. Receiver operator characteristic (ROC) curve for EPD diagnosis (FE-1 ≤100mcg/g) based on the multivariate logistic regression from Table 3, A) with PERT use variable, and B) without PERT use variable.

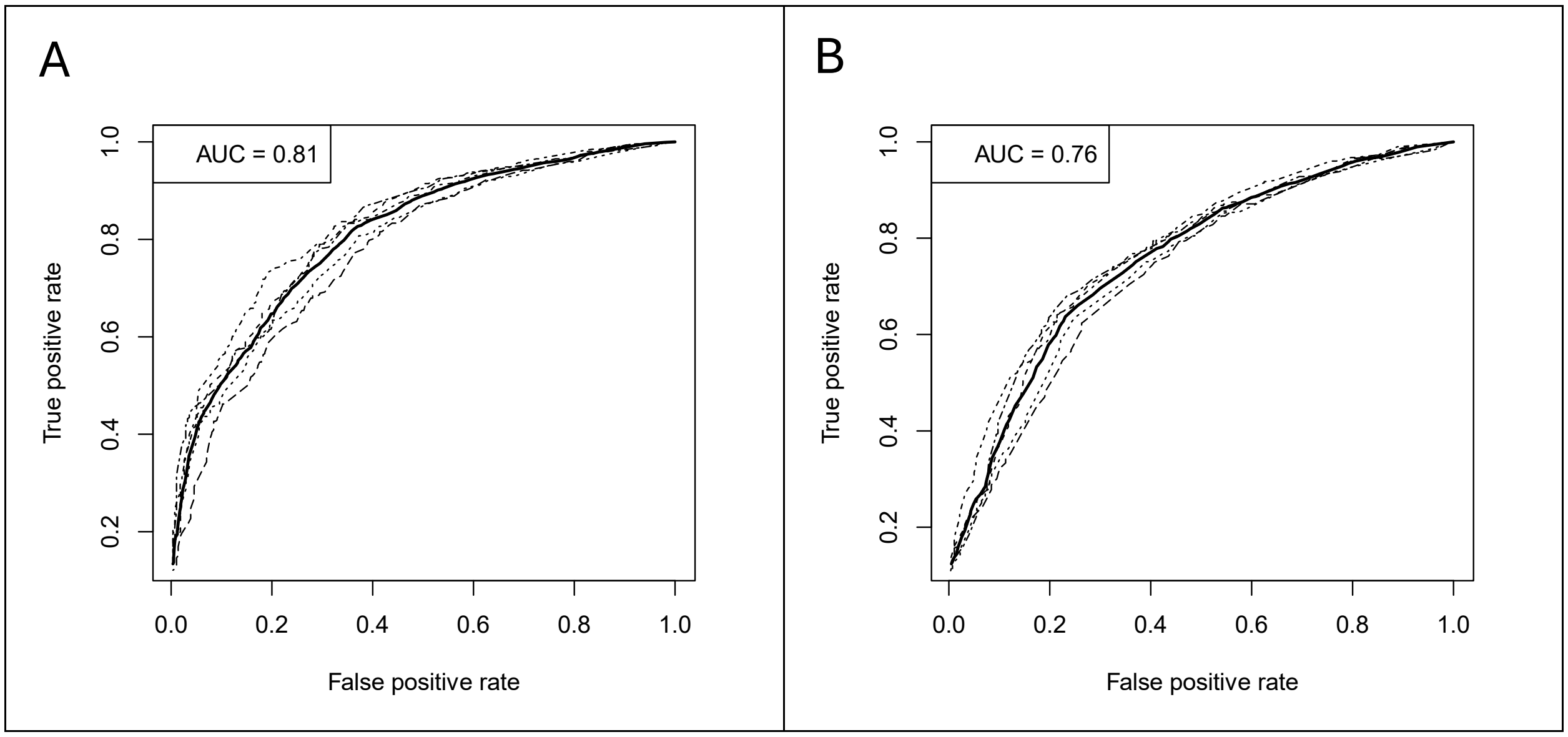

Supplement: Supplementary file 1 [file mmc1.pdf]
