# Supplementary material for: Exocrine Pancreatic Dysfunction in Chronic Pancreatitis: A Cross-Sectional Analysis of the Prospective Evaluation of Chronic Pancreatitis for Epidemiologic and Translational Studies Study (PROCEED)
Source: Gastro Hep Adv. 2026 Sep 24;5(12):101121. doi: 10.1016/j.gastha.2026.101121 (PMC13757507; doi:10.1016/j.gastha.2026.101121)

Gastro Hep Advances 2026;5:101121

ORIGINAL RESEARCH—CLINICAL

Exocrine Pancreatic Dysfunction in Chronic Pancreatitis: A Cross-Sectional Analysis of the Prospective Evaluation of Chronic Pancreatitis for Epidemiologic and Translational Studies Study (PROCEED)

Mitchell L. Ramsey,[1] Dhiraj Yadav,[2] Peng Yang,[3,4] Liang Li,[4] Melena Bellin,[5] William E. Fisher,[6] Evan L. Fogel,[7] Christopher E. Forsmark,[8] Phil A. Hart,[1] Walter G. Park,[9] Stephen J. Pandol,[10] Temel Tirkes,[11] Mark Topazian,[12] Stephen Van Den Eeden,[13] Santhi Swaroop Vege,[13] Radmila Choate,[14] Rachel L. Hill,[15] and Darwin L. Conwell,[15] on Behalf of the Consortium for the Study of Chronic Pancreatitis, Diabetes, and Pancreatic Cancer (CPDPC)

[1]Division of Gastroenterology, Hepatology, and Nutrition, College of Medicine, The Ohio State University Wexner Medical Center, Columbus, Ohio; [2]Division of Gastroenterology, Hepatology, and Nutrition, University of Pittsburgh Medical Center, Pittsburgh, Pennsylvania; [3]Department of Statistics, Rice University, Houston, Texas; [4]Department of Biostatistics, University of Texas MD Anderson Cancer Center, Houston, Texas; [5]Division of Pediatric Endocrinology, University of Minnesota, Minneapolis, Minnesota; [6]Department of Surgery, Baylor College of Medicine, Houston, Texas; [7]Division of Gastroenterology and Hepatology, Indiana University, Indianapolis, Indiana; [8]Division of Gastroenterology, Hepatology, and Nutrition, University of Florida, Gainesville, Florida; [9]Division of Gastroenterology, Hepatology, and Nutrition, Stanford University, Palo Alto, California; [10]Division of Gastroenterology, Hepatology, and Nutrition, Los Angeles, California; [11]Department of Radiology and Imaging Sciences, Indiana University, Indianapolis, Indiana; [12]Division of Gastroenterology, Hepatology, and Nutrition, Mayo Clinic, Rochester, Minnesota; [13]Division of Gastroenterology, Hepatology, and Nutrition, Kaiser Permanente Division of Research, Oakland, California; [14]Department of Epidemiology and Biostatistics, College of Public Health, University of Kentucky, Lexington, Kentucky; and [15]Division of Gastroenterology, Hepatology, and Nutrition, University of Kentucky, Lexington, Kentucky

BACKGROUND AND AIMS: Changes in exocrine function are believed to be common and associated with morbidities in chronic pancreatitis (CP), but there are many limitations with previous research to characterize this issue. This study aimed to determine the prevalence of and associations for exocrine pancreatic dysfunction (EPD) in a well-characterized CP study population. **METHODS:** We analyzed data at enrollment from participants with definite CP enrolled in Prospective Evaluation of Chronic Pancreatitis for Epidemiologic and Translational Studies, an ongoing multicenter study. Participants with no known EPD prior to enrollment (ie, no clinical steatorrhea or normal testing) were offered fecal elastase-1 (FE-1) testing. EPD was defined using FE-1 ≤100 mcg/g (primary) and <200 mcg/g stool (secondary) cutoffs. Multiple imputation estimated EPD prevalence in those who did not undergo FE-1 testing. Multivariable regression identified patient- and disease-related correlates of EPD. **RESULTS:** Among 681 participants, EPD point prevalence was 59.4% using <100 mcg/g and 70% using <200 mcg/g cutoff. Among 419 with assessed EPD status, EPD (<100 mcg/g) independently associated with diabetes (odds ratio [OR] 1.91 [1.10, 3.32]), alcohol etiology (OR 2.13 [1.10, 4.11]), pancreatic calcifications (OR 2.96 [1.67, 5.24]), atrophy (OR 3.23 [1.38, 7.55]), pancreatic enzyme replacement therapy (OR 4.67 [2.84, 7.65]), and Charlson Comorbidity Index ≥2 (OR 3.33 [1.08, 10.22]). EPD prevalence was higher in those with low/normal body mass index (vs overweight/obese) and among participants with diabetes across all body mass index categories. Using the secondary cutoff, the associations for EPD were generally similar, and these participants reported significantly reduced physical function and social participation. **CONCLUSION:** EPD is common in CP and is associated with multiple clinical features and changes in pancreatic morphology. Future evaluation should further assess the impact of EPD on nutritional parameters at initial diagnosis and during the disease course, and its response to pancreatic enzyme replacement therapy.

Keywords: Exocrine Pancreatic Dysfunction (EPD); Fecal Elastase-1; Steatorrhea; Malabsorption

Introduction

Chronic pancreatitis (CP) is a progressive, fibroinflammatory disorder that leads to the destruction of

Abbreviations used in this paper: **AP, acute pancreatitis; AUC, area under the receiver operating characteristic curve; CCI, Charlson Comorbidity Index; CP, chronic pancreatitis; DXA, dual-energy X-ray absorptiometry; EPD, exocrine pancreatic dysfunction; FE-1, fecal elastase-1; OR, odds ratio; PERT, pancreas enzyme replacement therapy; PROCEED, Prospective Evaluation of Chronic Pancreatitis for Epidemiologic and Translational Studies; QoL, quality of life; ROC, receiver operating characteristic.**

Most current article

© 2026 The Authors. Published by Elsevier Inc. on behalf of American Gastroenterological Association Institute. This is an open access article under the CC BY license (http://creativecommons.org/licenses/by/4.0/).
2772-5723
https://doi.org/10.1016/j.gastha.2026.101121

the pancreatic parenchyma, causing both endocrine and exocrine dysfunction.[1] Exocrine pancreatic insufficiency (EPI) can involve a range of symptoms and long-term sequelae. Severe EPI often presents with gastrointestinal symptoms of maldigestion (steatorrhea) and can lead to weight loss, malnutrition, and fat-soluble vitamin deficiencies.[2,3] Mild to moderate cases of EPI, although at risk for complications of malabsorption, may go undetected due to nonspecific symptoms and limited sensitivity of currently available laboratory tests.[4,5] Recognition of the prevalence of EPI along the CP disease spectrum remains a significant research knowledge gap.

Fecal elastase-1 (FE-1) has emerged as the most widely used clinical tool to assess for EPI due to its simplicity and noninvasive nature.[6,7] The use of FE-1 in various clinical situations has been well-described, and a recent systematic review demonstrates that a low FE-1 value has a high positive predictive value for exocrine pancreatic dysfunction (EPD) among individuals with CP.[7] However, because FE-1 does not directly measure fat malabsorption, The Chronic Pancreatitis Working Group from of the Consortium for the Study of Chronic Pancreatitis, Diabetes, and Pancreatic Cancer adopted the term "exocrine pancreatic dysfunction" to describe the spectrum of functional decline which can be measured with FE-1 in the presence or absence of fat malabsorption.[2] This nomenclature encompasses a broader range of reduced exocrine function, including those with mild dysfunction and severe dysfunction (ie, EPI) and is intended to support a more nuanced understanding of disease progression in CP.

Prospective Evaluation of Chronic Pancreatitis for Epidemiologic and Translational Studies (PROCEED) is a multicenter, longitudinal cohort study designed to investigate the natural history of CP.[8] Participants with CP without clinically diagnosed EPI at enrollment are offered routine FE-1 testing at enrollment and during follow-up to systematically estimate the exocrine pancreatic function. PROCEED thus provides a unique research opportunity, leveraging its robustly collected patient-level and disease-level data to investigate factors associated with EPD status in CP.

The primary aim of this analysis was to determine the point prevalence of EPD at enrollment into PROCEED. The secondary aim was to explore the association of EPD with patient- and disease-related features. Furthermore, we also explored the association of EPD with clinical consequences of EPD, such as poor bone health, bone fractures, serum vitamin D levels, and quality of life (QoL). By incorporating both a per-protocol FE-1 assessment and multiple imputation methods into our study design, we sought to more fully characterize the prevalence of EPD in CP.

Methods

Study Population

Participants in the PROCEED study (NCT03099850) were recruited from 9 US clinical centers of the Chronic Pancreatitis, Diabetes, and Pancreatic Cancer.[8,9] The central institutional review board at MD Anderson as well as the institutional review board at each clinical center reviewed and approved the study. The current analysis includes 681 participants with definite CP enrolled between June 2017 and November 2021. Definite CP was defined by parenchymal or ductal calcifications, Cambridge classification of 3 or 4 on CT scan and or magnetic resonance imaging (MRI), or by histology.[8,10] Imaging studies were reviewed by abdominal radiologists at each center using a standardized data collection form and reporting standards.[10,11]

Definitions

Exocrine pancreatic dysfunction. As shown in Figure 1, participants with known EPD (clinical signs of steatorrhea, quantitative [24 h] stool fat excretion of >7 g (g) or FE-1 of ≤ 100 μg (mcg)/g stool) prior to enrollment were considered to have EPD. Participants with no known EPD at enrollment were offered routine FE-1 testing, and the results were used to categorize EPD status. Overall, complete information on EPD status was available in 419/681 (62%) of participants — they are referred to as the "Assessed Cohort" (Figure 1) and all primary analyses were performed in these participants. Per-protocol FE-1 testing was not completed by 262 participants, so EPD status in these participants was determined by multiple imputation (see Statistical Analysis section), and this group is referred to as the "Imputed Cohort." Ultimately, the EPD status of all participants was established and secondary analyses were performed in all participants referred to as the "Full Cohort" (Full Cohort = Assessed Cohort + Imputed Cohort).

Two cutoff points were used to classify EPD: the first cutoff defined EPD by participants having known EPD prior to enrollment or per-protocol FE-1 values of <100 mcg/g. This cutoff of 100 mcg/g stool was used due to increased specificity. For the second cutoff, EPD was defined by participants having known EPD prior to enrollment or per-protocol FE-1 values of <200 mcg/g — this cutoff was used because it is more widely used clinically and in the published literature.[12]

Diabetes. Diabetes status was assessed at enrollment using the American Diabetes Association diagnostic criteria.[13] Similar to EPD, diagnosis of diabetes prior to enrollment was noted — diabetes diagnosis was confirmed when a participant had abnormal values on 2 of the following tests or 2 abnormal values of the same test (fasting blood glucose ≥ 126 mg/dl, hemoglobin A1c $\geq 6.5\%$, random blood sugar ≥ 200 mg/dl) or treatment with antidiabetic medication(s). Participants with no known diabetes diagnosis prior to enrollment were offered to undergo per-protocol assessment with blood glucose and hemoglobin A1c measurements. Thus, diabetes status at enrollment could be assessed for nearly all individuals (95.3%), and multiple imputation was performed for the remaining participants.

Clinical Data

Clinical data were collected through a combination of self-administered case report forms, direct interviews of participants conducted by trained clinical research coordinators, and a thorough review of participants' electronic health records. Variables of interest to evaluate for associations with EPD included demographics, socioeconomic status, smoking and

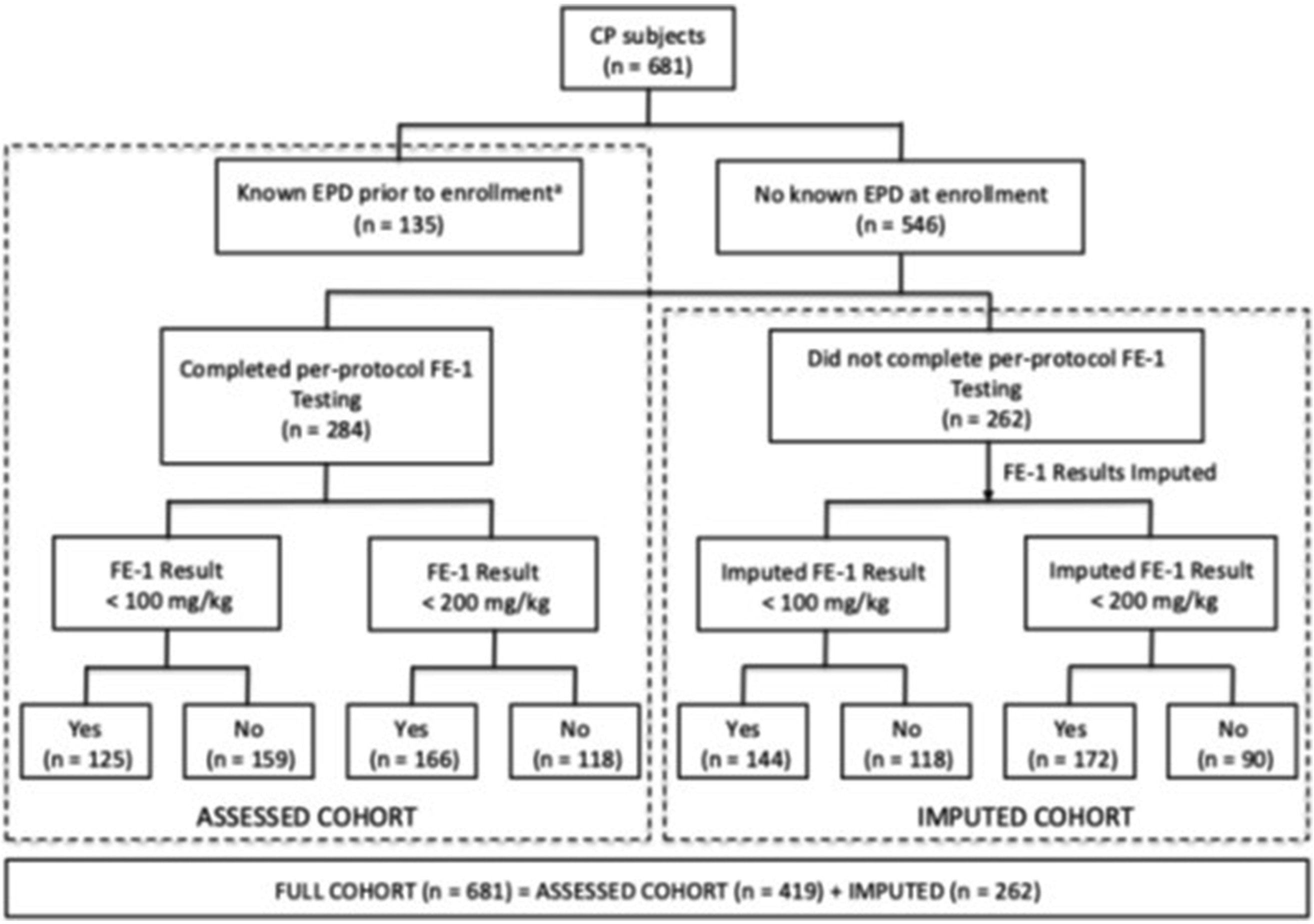

Figure 1. Exocrine pancreas dysfunction (EPD) study flow diagram. [a]Method of EPD diagnosis: Clinical signs (eg, steatorrhea), n = 61; FE-1 <100, n = 84; fecal fat >7 gm, n = 7; unknown, n = 3. See PROCEED definitions in the Methods section.

drinking status (categorized as never, past, current), and Charlson Comorbidity Index (CCI) Category. CCI Category was defined as follows: Category 1 (CCI score 0-1); Category 2 (CCI score 2-3); and Category 3 (CCI >3), which is a weighted score reflecting the number and severity of comorbid conditions. Disease-specific features included etiology, prior history of acute pancreatitis (AP), duration of pancreatitis, imaging evidence of calcifications and pancreas size, pancreatic enzyme replacement therapy (PERT), and endoscopic therapy or pancreatic surgery. Etiology of CP was categorized as alcohol, idiopathic, or other as adjudicated by the clinical center investigators. Additional variables were self-reported pain severity and temporal nature in the year prior to enrollment.

Potential consequences of EPD that could be further explored in PROCEED include bone health based on dual-energy X-ray absorptiometry (DXA) scan, history of bone fractures, QOL as assessed by Patient-Reported Outcomes Measurement Information System (PROMIS) Global Health instrument, and vitamin D levels. Information on DXA scans performed within 3 years prior to enrollment was recorded; those with no DXA or if performed >3 years prior to enrollment were offered to undergo a per-protocol DXA scan. DXA scan results were classified as normal, osteopenia, or osteoporosis. Information on bone fractures was noted and organized by severity as minor, moderate, or major.[14] Participant responses to PROMIS Global Health instrument were used to generate T-scores for Physical and Mental Health from PROMIS 29 for 7 domains spanning physical, mental, and social health using suggested scoring algorithm.[15,16]

Statistical Analyses

The primary outcome of interest was the point prevalence of EPD at enrollment. Comparisons between participants with and without EPD aimed to identify patient- and disease-related features that associate with EPD. We also evaluated whether consequences that may be related to EPD are more common among participants with EPD. For univariate analysis, we summarized continuous data by median and interquartile range and categorical variables by counts and percentages. We applied Wilcoxon rank sum test for continuous variables and the Fisher exact test for categorical variables, and report the corresponding *P* values. Missing values were excluded from the two-sample tests, with missing value counts reported in the tables as a superscript. All statistical analyses were conducted in R (www.r-project.org), with *P* value <.05 considered statistically significant.

We performed multiple imputations with the "mice" package in R to impute missing values among the variables used in the multivariable analysis, particularly missing EPD status due to noncompliance with per-protocol FE-1 testing, in order to determine a point prevalence within the Full Cohort. The imputation was conducted under the full conditional

Table 1. Prevalence of EPD at Baseline Assessment Among Definite CP Participants in PROCEED According to EPD Diagnosis Prior to Enrollment or by per Protocol FE-1 Assessment (Assessed Cohort), or Imputed EPD Status (Full Cohort)

Point prevalence of EPD	Assessed cohort (n = 419)	Full cohort (n = 681)
Known EPD prior to enrollment or FE-1 <100 mcg/g stool	260 (62.1%)	404 (59.3%)
Known EPD prior to enrollment or FE-1 <200 mcg/g stool	301 (71.8%)	473 (69.5%)

specification framework, which models each variable with missing data conditionally on all other variables. Predictive mean matching, logistic regression, and proportional odds models were applied for continuous, categorical, and ordered variables, respectively, when missing values occurred. Each of the 5 imputed datasets went through a stepwise model selection procedure to identify a parsimonious model with significant predictors. Predictors selected in more than 3 imputed datasets were retained in the final model. The parameters of the final model were estimated in a combined analysis of all 5 imputed datasets, with the final selected predictors.

Multivariable logistic regression analysis was performed in the Assessed Cohort (n = 419) and separately in the Full Cohort (n = 682) where the outcome was EPD (vs no EPD) and the patient- and disease-related variables were predictors. We first performed the analysis defining EPD according to FE-1 <100 mcg/g, and subsequently repeated the analysis using the alternate definition of FE-1 <200 mcg/g. Similarly, the analyses were repeated with and without the PERT use variable, as PERT is used to treat EPD and might confound associations with other covariates of interest. We assessed the final model's goodness of fit using the receiver operating characteristic (ROC) curve and the area under the curve (AUC). These measures quantify the overall association between the selected predictors and EPD.

T-scores for each PROMIS-29 domain were compared between participants with and without EPD according to both definitions (FE-1 <100 mcg/g and FE-1 <200 mcg/g) and displayed using radar charts.

Results

Study Population

Among the 681 participants with CP (Full Cohort), 53.5% were male and 83.0% were white (data not shown). The median age at enrollment was 55 years, with a median body mass index (BMI) of 25. The median duration of CP was 3 years. Approximately 36% of participants had education beyond high school, over 50% were married, and 40% were currently employed. Current smoking and drinking were reported by 37% and 23% of participants, respectively. The most common etiologies for pancreatitis were alcohol (42%) and idiopathic (38%). A prior history of AP was noted in 81% of subjects. Diabetes mellitus was present in 42%. Endoscopic therapy had been received by 54% of participants, while 9% had undergone prior pancreatic surgery. The majority of participants (75%) had advanced morphologic changes on imaging (ie, calcifications and/or parenchymal atrophy).

As shown in Figure 1, of the 681 participants, 135 had known EPD prior to enrollment. The remaining 546 participants with no known EPD were offered per-protocol FE-1 testing. Of these, 284 (52.0%) underwent FE-1 testing and complete the Assessed Cohort, while 262 participants (47.9%) did not have FE-1 testing (Imputed Cohort). Thus, the Full Cohort (n = 681) consisted of the Assessed (n = 419) and Imputed (262) cohorts. Participants in the Assessed Cohort were generally similar to the Imputed Cohort, with the exception that the Imputed Cohort were younger (median age at enrollment 54 vs 52, $P < .01$), had a higher proportion of active smokers (44% vs 33%, $P < .01$), and had a higher proportion of alcohol etiology (46% vs 39%, $P = .02$) (data not shown).

Point Prevalence of Exocrine Pancreatic Dysfunction and Univariate Analyses — Assessed Cohort

Overall, the point prevalence of EPD in the Assessed Cohort was similar to the Full Cohort at both cutoff definitions: FE-1 < 100 mcg/g (62% vs 59%) and FE-1 < 200 mcg/g (72% vs 70%) (Table 1). Among the Assessed Cohort, those with FE-1 <100 had lower BMI, longer AP and CP disease duration, more advanced morphologic changes, and a higher frequency of current smokers and participants with diabetes than those with FE-1 >100 mcg/g (Table 2). There were differences in the social determinants of health with individuals with FE-1 <100 mcg/g demonstrating lower education level, lower income, and higher rates of unemployment or disability. These results were generally similar when the secondary FE-1 <200 mcg/g cutoff was used (Table 2), and the subset of individuals with FE-1 results between 100 and 200 mcg/g were generally similar to the >200 mcg/g group (Supplementary Table 1).

Multivariable Analyses — Assessed Cohort

Table 3 shows multivariate logistic regression models with and without inclusion of PERT use as a covariate. Using FE-1 <100 mcg/g cutoff, PERT use was the strongest factor associated with EPD. When compared with participants without EPD, the odds of using PERT were 4.67 times greater in participants with EPD. The other significant associations were with diabetes, alcohol etiology, morphologic features on imaging, endoscopic therapy, and presence of comorbidities. In the model without PERT use, the direction of associations for other factors was similar. Using these variables, the ROC curve with and without

Table 2. Baseline Characteristics of CP Participants With and Without EPD in Whom EPD Status Was Assessed Prior to or at Enrollment (Assessed Cohort, n = 419)

Variable	FE-1 <100 (n = 260)	FE-1 ≥100 (n = 159)	*P* value	FE-1 <200 (n = 301)	FE-1 ≥200 (n = 118)	*P* value
Demographics						
Median age at enrollment, y; median (IQR)	56 (46–63)	55 (45–65)	.90	56 (46–63)	57 (45–65)	.58
Female sex, *n* (%)	114 (43.8)	83 (52.2)	.11	134 (44.5)	63 (53.3)	.10
White race, *n* (%)	215 (82.7)	140 (88.1)	.12	254 (84.4)	101 (85.6)	.88
BMI, kg/m^2; median (IQR)	23.9 (21.0–28.0)	25.2 (21.7–29.1)	.03	24.1 (21.0–28.0)	25.2 (21.8–29.3)	.05
Social determinants of health						
Highest level of education, *n* (%)						
High school, General Equivalency Diploma (GED), or less	90 (34.9)[2]	47 (30.1)[3]	.46	103 (34.6)[3]	34 (29.3)[2]	.21
Something training past HS	88 (34.1)	52 (33.3)		104 (34.9)	36 (31.0)	
Bachelor's or graduate school	80 (31.0)	57 (36.5)		91 (30.5)	46 (39.7)	
Family income, *n* (%)						
Less than 50 k	119 (49.8)[21]	50 (34.5)[14]	<.01	130 (47.4)[27]	39 (35.5)[8]	.04
50–100 k	67 (28.0)	42 (29.0)		78 (28.5)	31 (28.2)	
100 k or greater	53 (22.2)	53 (36.6)		66 (24.1)	40 (36.4)	
Marital status, *n* (%)						
Single	59 (22.8)[1]	32 (20.1)	.23	67 (22.3)[1]	24 (20.3)	.87
Married	140 (54.1)	99 (62.3)		169 (56.3)	70 (59.3)	
Divorced/separated/widowed	60 (23.2)	28 (17.6)		64 (21.3)	24 (20.3)	
Employment status, *n* (%)						
Unemployed	97 (37.9)[4]	37 (23.9)[4]	.01	109 (36.9)[6]	25 (21.6)[2]	<.01
Employed	95 (37.1)	72 (46.5)		112 (38.0)	55 (47.4)	
Retired	64 (25.0)	46 (29.7)		74 (25.1)	36 (31.0)	
Disability or medical leave, *n* (%)	89 (35.6)[10]	33 (23.6)[17]	.02	99 (34.5)[14]	23 (22.3)[13]	.03
Clinical characteristics						
Smoking status, *n* (%)						
Current	98 (37.8)[1]	41 (25.9)[1]	.03	109 (36.3)[1]	30 (25.6)[1]	.04
Past	78 (30.1)	51 (32.3)		94 (31.3)	35 (29.9)	
Never	83 (32.0)	66 (41.8)		97 (32.3)	52 (44.4)	
Charlson Comorbidity Index category, *n* (%)						
1	159 (61.2)	132 (83.0)	<.01	196 (65.1)	95 (80.5)	<.01
2	79 (30.4)	18 (11.3)		81 (26.9)	16 (13.6)	
3	22 (8.5)	9 (5.7)		24 (8.0)	7 (5.9)	
Diabetes, *n* (%)	138 (55.0)[9]	41 (27.9)[12]	<.01	150 (52.2)[14]	29 (26.1)[7]	<.01
PERT usage, *n* (%)	184 (71.0)[1]	45 (28.8)[3]	<.01	200 (66.9)[2]	29 (25.0)[2]	<.01
Prior AP, *n* (%)	206 (82.7)[11]	134 (85.4)[2]	.58	240 (83.0)[12]	97 (85.5)[1]	.66
Duration of pancreatitis prior to enrollment, yrs, median (IQR)	5 (2–11)	3 (1–11)[1]	.02	5 (2–11)	3 (1–11)[1]	.06
Etiology, *n* (%)						
Alcohol	126 (48.5)	38 (23.9)	<.01	140 (46.5)	24 (20.3)	<.01
Idiopathic	92 (35.4)	76 (47.8)		106 (35.2)	62 (52.5)	
All others combined	42 (16.2)	45 (28.3)		55 (18.3)	32 (27.1)	
Pancreatic calcifications, *n* (%)	221 (85.0)	93 (58.5)	<.01	244 (81.1)	70 (59.3)	<.01
Pancreas size on imaging, *n* (%)						
<7 mm	52 (20.0)	10 (6.3)[1]	<.01	55 (18.3)	7 (6.0)[1]	<.01
7–14 mm	117 (45.0)	50 (31.6)		139 (46.2)	28 (23.9)	
>14 mm	91 (35.0)	98 (62.0)		107 (35.5)	82 (70.1)	
Endoscopic therapy, *n* (%)	145 (56.4)[3]	71 (45.5)[3]	.03	169 (56.9)[4]	47 (40.5)[2]	<.01
Pancreatic surgery, *n* (%)	35 (13.5)[1]	8 (5.0)	<.01	39 (13.0)[1]	4 (3.3)	<.01

Number of participants with missing data is annotated in superscript.
IQR, interquartile range.

PERT use variable produced an area under the curve (AUC) of 0.81 and 0.76, respectively (Supplementary Figure 1A and B). The associations with EPD were generally similar when using FE < 200 mcg/g as the cutoff, although comorbidities and endoscopic therapy were no longer independently associated with EPD. The associations with EPD were generally similar in the Full Cohort (data not shown).

Since BMI did not meet the cutoff for inclusion in multivariable models, we evaluated the prevalence of EPD in different BMI categories and presence of diabetes (Figure 2). The prevalence of EPD was higher in participants with diabetes across all BMI categories, and the prevalence of EPD was inversely correlated with BMI.

Table 3. Multivariable Logistic Regression Using Stepwise Selection Showing Factors Independently Associated With the Presence of EPD Among Participants With CP in the Assessed Cohort (n = 419)

Variables: Category	Reference	OR (95% CI) With PERT use variable	*P* value With PERT use variable	OR (95% CI) Without PERT use variable	*P* value Without PERT use variable
EPD defined as FE-1 <100 mcg/g					
Intercept		0.09 (0.03, 0.31)	<.01	0.21 (0.07, 0.64)	.01
Diabetes: Yes	No	1.91 (1.10, 3.32)	.02	2.03 (1.20, 3.43)	.01
PERT usage: Yes	No	4.67 (2.84, 7.65)	<.01	n/a	n/a
Etiology of CP	All other				
Idiopathic		0.91 (0.49, 1.70)	.77	0.93 (0.51, 1.67)	.80
Alcohol-related		2.13 (1.10, 4.11)	.02	2.49 (1.33, 4.66)	<.01
Calcification: Yes	No	2.96 (1.67, 5.24)	<.01	2.89 (1.70, 4.93)	<.01
Atrophy	>14 mm				
7–14 mm		1.70 (1.02, 2.85)	.04	1.94 (1.19, 3.15)	.01
<7 mm		3.23 (1.38, 7.55)	.01	3.27 (1.51, 7.48)	<.01
Charlson Comorbidity Index (CCI)	CCI = 3				
CCI 1		1.31 (0.48, 3.61)	.60	1.02 (0.40, 2.57)	.97
CCI 2		3.33 (1.08, 10.22)	.04	2.54 (0.90, 7.17)	.08
Endoscopic therapy: Yes	No	2.23 (0.87, 5.73)	.01	2.90 (1.19, 7.02)	.02
EPD defined as FE-1 <200 mcg/g					
Intercept		0.31 (0.16, 0.59)	<.01	0.51 (0.28, 0.93)	.03
Diabetes: Yes	No	2.30 (1.33, 3.98)	<.01	3.29 (1.94, 5.58)	<.01
PERT usage: Yes	No	4.57 (2.71, 7.70)	<.01	n/a	n/a
Etiology of CP	All other				
Idiopathic		0.82 (0.44, 1.52)	.52	0.83 (0.46, 1.51)	.55
Alcohol-related		2.28 (1.13, 4.63)	.02	2.76 (1.40, 5.42)	<.01
Calcification: Yes	No	1.74 (1.30, 3.04)	.05	1.80 (1.06, 3.06)	.03
Atrophy	>14 mm				
7–14 mm		3.01 (1.73, 5.23)	<.01	3.29 (1.94, 5.58)	<.01
<7 mm		3.52 (1.41, 8.84)	.01	4.00 (1.65, 9.70)	<.01

CI, confidence interval.

Consequences of Exocrine Pancreatic Dysfunction

Within the Assessed Cohort, we performed an exploratory analysis of the consequences of EPD, including bone health and QOL measures. Only a subset had available data for DXA scan (239/419) or vitamin D levels (228/419), and 80 and 229 were already receiving vitamin D replacement and PERT at the time of enrollment into PROCEED. With these limitations, we did not observe a difference in the prevalence of osteopathy, fractures, or vitamin D levels using either FE-1 cutoff (Supplementary Table 2). The T-scores for the PROMIS 29 domains showed no difference between those with or without EPD when analysis was performed using FE-1 <100 mcg/g cutoff. At FE-1 <200 mcg/g, participants with EPD reported significantly impaired physical function and reduced engagement in social roles and activities (Figure 3).

Discussion

In a cohort of well-characterized patients with CP, we demonstrate a high point prevalence of EPD. Although PERT use was the strongest factor associated with EPD, many patients with documented EPD were not receiving enzyme replacement and effect sizes for other risk factors remained similar when PERT use was not included. Other clinical factors associated with EPD were alcohol etiology, diabetes, and imaging evidence of advanced disease. In an exploratory analysis, we found an association of EPD with impaired physical function and participation in social roles and activities. PERT is recommended to prevent long-term consequences of malabsorption. Therefore, EPD should be tested in all patients with CP; a positive test among patients with certain clinical features (eg, alcohol etiology, diabetes, pancreatic calcifications) is expected to have a high positive predictive value for EPD.

While many studies use robust criteria to identify EPD as present (eg, abnormal FE, clinical steatorrhea, etc.), the methods for ruling out EPD may introduce bias in determining the true point prevalence of EPD in CP. For example, some studies have defined EPI according to PERT use, which underestimates the prevalence of EPI by likely missing untreated mild or moderate cases and may introduce false positives for patients empirically treated with PERT for symptom management.[17] Another strategy often employed is to include only individuals who completed some objective testing for EPD.[18,19] However, this strategy risks selection bias as only symptomatic individuals will be offered and complete stool-based tests, and therefore may overestimate the prevalence of EPD. Rather, uniformly

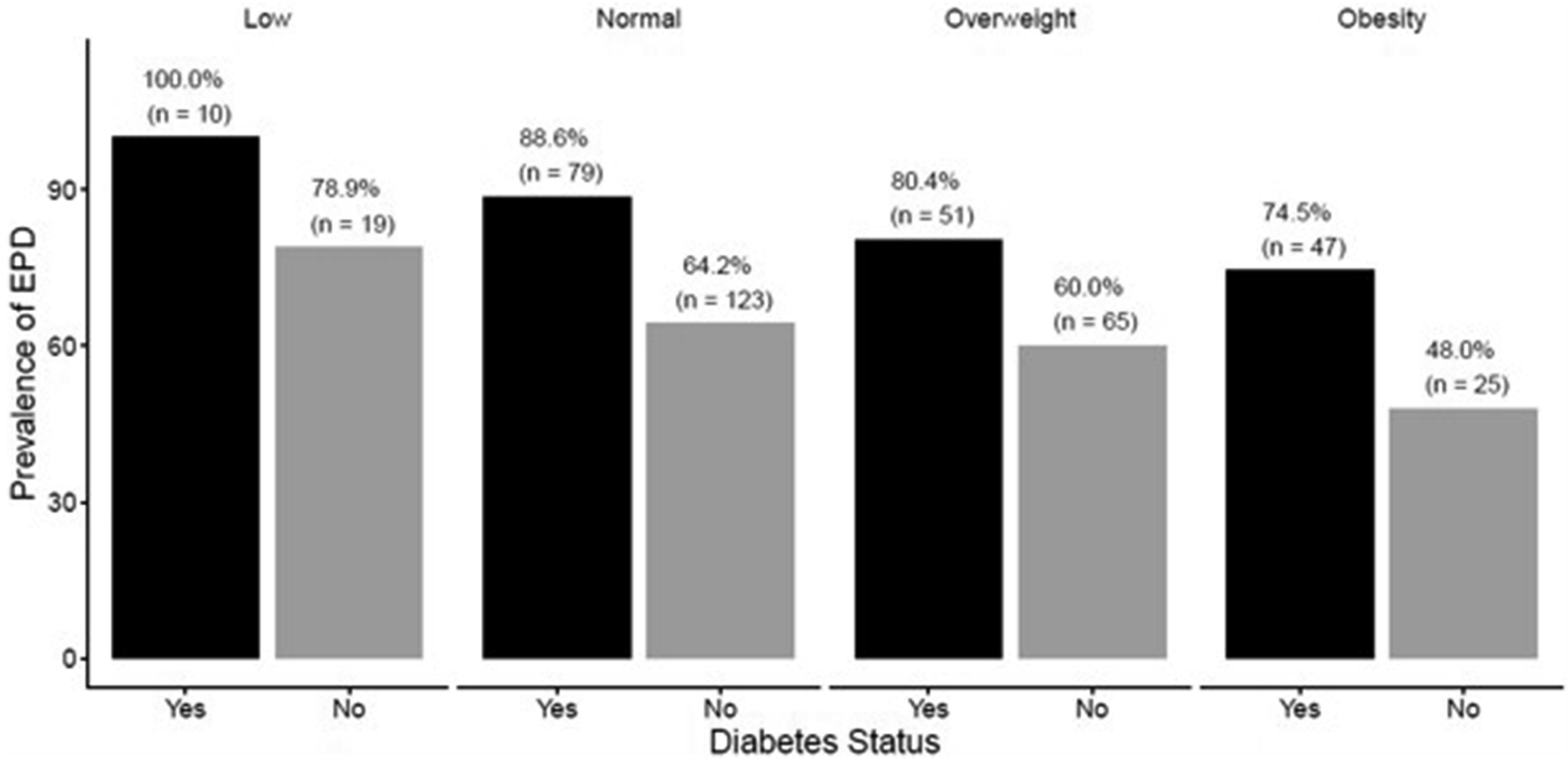

Figure 2. Prevalence of EPD at baseline assessment among definite CP participants in PROCEED with Assessed EPD Status according to BMI category among participants with and without diabetes (n = 179 and 219, respectively).* *Prevalence uses the Assessed EPD cohort, with EPD defined by FE-1 <100 mcg/g. Prevalence of each column is calculated as the number of subjects with EPD out of the number of subjects with the same BMI category and diabetes status. Diabetes status was not tested for 2, 8, 8, and 3 individuals in the low, normal, overweight, and obesity categories, respectively.

offering stool based testing regardless of gastrointestinal symptoms should minimize selection bias and provide a more accurate representation of EPD status in the population. PROCEED represents the first large-scale study to implement a routine, systematic assessment of EPD in CP. While approximately one-third of patients did not undergo per-protocol testing, the use of multiple imputation provided a robust estimate of EPD prevalence across the entire cohort. Follow-up FE-1 testing during the PROCEED study will allow for longitudinal evaluation, further refining our understanding of the natural history of EPD in CP.

The diagnosis of EPD can be straightforward for individuals with severe EPD based on symptoms (ie, steatorrhea), clinical signs (ie, weight loss, vitamin deficiencies), and clinical history (eg, history of severe AP, longstanding CP). In contrast, objective testing is often required to

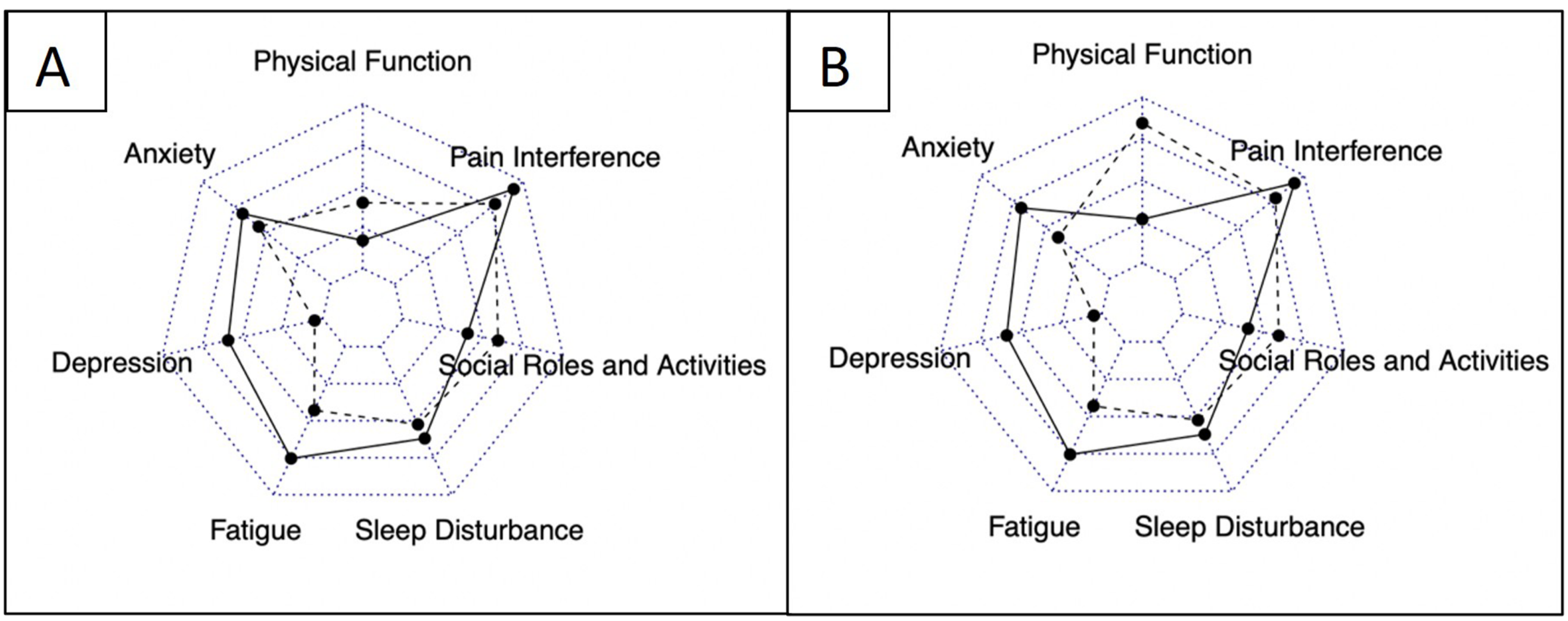

Figure 3. Radar charts illustrating the comparison of PROMIS-29 domain scores for definite CP participants in the Assessed Cohort (n = 419) with and without EPD according to the definition of EPD; (A) FE-1 <100 mcg/g and (B) FE-1 <200 mcg/g. The solid and dashed lines indicate with and without EPD, respectively.

diagnose individuals with mild to moderate EPD since symptoms can be unreliable in this clinical scenario.[4] The current "clinical standard" for a diagnosis of EPD is the FE-1 test, although this only measures exocrine capacity and does not assess the degree of fat malabsorption or efficacy of PERT.[2,6] Additionally, interpretation of the FE-1 result requires an understanding of the test performance characteristics integrated with the patient's risk profile to determine the posttest probability of EPD.[7] Accordingly, the optimal cutoff threshold for EPD remains a matter of debate, with FE-1 <200 mcg/g as a viable threshold when used for screening, and <100 mcg/g (or lower) when greater specificity is required.[2,7] Our study demonstrates that alcohol etiology, endocrine dysfunction, and imaging evidence of advanced CP associate with EPD, which provide a helpful framework for assessing the pre-test probability of EPD and may provide a rationale for clinicians when counseling patients on the importance of stool sample submission.

In this study, we investigated clinical features that are associated with the presence of EPD. Based on prior reports, pancreatic structural changes, diabetes, disease duration, and alcohol etiology have been strongly associated with the presence of EPD.[18–21] Similarly, we found imaging features of advanced disease (calcifications, atrophy), diabetes, and alcohol etiology to be significantly associated with the presence of EPD. These features had modest performance for the diagnosis of EPD according to FE-1 ≤100 mcg/g when plotted on ROC curve (AUC 0.76) (Supplementary Figure 1). Importantly, BMI was not significantly associated with EPD in the multivariable model and participants across all BMI categories could be found to have EPD. Clinicians may consider each of these features to increase the suspicion for EPD; however, there remains a need for objective testing to confirm a diagnosis and initiate therapy. Thus, clinical features are useful for suggesting a diagnosis of EPD, but there remains a need for objective biomarkers beyond clinical features to accurately diagnose and treat EPD.

The impact of EPD on QOL and metabolic bone disease remains complex. While we observed associations with some QOL measures at the ≤200 mcg/g cutoff, the same did not hold true at the ≤100 mcg/g cutoff. This is likely due to a smaller sample size in the ≤100 mcg/g cohort. Additionally, given that two-thirds of our cohort was already receiving PERT, the full impact of untreated EPD could not be fully assessed in this study. A future direction of our study will be to stratify patients based on treatment status to provide a clearer picture of the burden of EPD on daily functioning and well-being. Similarly, our study did not identify a significant association between EPD and bone health, but this finding is limited because only a subset of participants completed a DXA scan. It is possible that those without DXA evaluation had different bone health than those who underwent assessment, introducing potential bias. Similarly, vitamin D levels were difficult to interpret, as many participants were on supplementation at the time of enrollment. A more definitive analysis would require evaluation of treatment-naïve patients to accurately assess the relationship between EPD and vitamin D deficiency.

Our study has several strengths, including a large-scale systematic assessment of EPD among a well-phenotyped cohort with CP. The multicenter design increases generalizability among referral centers, including varied patient demographics and physician practice patterns, although our findings may not necessarily be generalizable to community centers. Our study also has several limitations, including the reliance on FE-1 as the primary diagnostic measure for EPD. While FE-1 is widely used in clinical practice, it has unavoidable limitations. Despite systematic reminders and the opportunity to complete the baseline FE-1 test at no cost, approximately one-third of participants did not submit a stool sample for analysis. We addressed this issue with missing data using multiple imputation methods that demonstrated the robustness of our findings. Additionally, the cross-sectional nature of our analysis and including many individuals on treatment (eg, PERT, vitamin replacement) limits causal interpretations, particularly as it relates to bone health and QOL measures, which may be impacted by short- or long-term treatments. Thus, our cross-sectional analysis may not be the appropriate study design for these outcomes and our findings on these outcomes should be considered exploratory. However, prior studies have shown similar results, with high levels of vitamin D deficiency and osteopathy regardless of PERT treatment.[22–25] Despite these limitations, our cohort meaningfully adds to the EPD literature by identifying a high-risk phenotype for EPD which can improve early recognition of EPD.

Conclusion

EPD is highly prevalent in 60%–70% of CP patients, particularly among those with structurally advanced pancreatic abnormalities, alcohol etiology, and diabetes. Improved diagnostic strategies for EPD are needed to capture more patients with earlier stages of CP without structural changes. Nevertheless, we recommend that objective exocrine function assessments (ie, FE-1) be integrated into the routine clinical care of all CP patients to facilitate early treatment. Future research should focus on refining diagnostic criteria, evaluating treatment-naïve populations, and integrating longitudinal data to assess disease progression and treatment response. Addressing these gaps will improve the recognition and management of EPD, ultimately enhancing patient outcomes in CP.

Supplementary Materials

Material associated with this article can be found, in the online version, at https://doi.org/10.1016/j.gastha.2026.101121.

References

1. Whitcomb DC, Frulloni L, Garg P, et al. Chronic pancreatitis: an international draft consensus proposal for a new mechanistic definition. Pancreatology 2016; 16:218–224.
2. Khan A, Vege SS, Dudeja V, et al. Staging exocrine pancreatic dysfunction. Pancreatology 2022; 22:168–172.
3. Ramsey ML, Hart PA, Forsmark CE. Evaluation and management of exocrine pancreatic insufficiency: pearls and pitfalls. Curr Opin Gastroenterol 2023; 39:428–435.
4. Durie P, Baillargeon JD, Bouchard S, et al. Diagnosis and management of pancreatic exocrine insufficiency (PEI) in primary care: consensus guidance of a Canadian expert panel. Curr Med Res Opin 2018;34:25–33.
5. Brownell JN, Schall JI, Stallings VA. Pancreatic function in chronic pancreatitis: a cohort study comparing 3 methods of detecting fat malabsorption and the impact of short-term pancreatic enzyme replacement therapy. Pancreas 2019;48:1068–1078.
6. Whitcomb D, Buchner A, Forsmark C. AGA clinical practice update on the epidemiology, evaluation, and management of exocrine pancreatic insufficiency: expert review. Gastroenterology 2023;165:1292–1301.
7. de la Iglesia D, Agudo-Castillo B, Galego-Fernández M, et al. Diagnostic accuracy of fecal Elastase-1 test for pancreatic exocrine insufficiency: a systematic review and meta-analysis. United Eur Gastroenterol J 2025; 13:1571–1582.
8. Yadav D, Park WG, Fogel EL, et al. PROspective evaluation of chronic pancreatitis for EpidEmiologic and translational StuDies: rationale and study design for PROCEED from the consortium for the study of chronic pancreatitis, diabetes, and pancreatic cancer. Pancreas 2018;47:1229–1238.
9. Serrano J, Andersen DK, Forsmark CE, et al. Consortium for the study of chronic pancreatitis, diabetes, and pancreatic cancer: from concept to reality. Pancreas 2018;47:1208–1212.
10. Tirkes T, Shah ZK, Takahashi N, et al. Inter-observer variability of radiologists for Cambridge classification of chronic pancreatitis using CT and MRCP: results from a large multi-center study. Abdom Radiol (NY) 2020; 45:1481–1487.
11. Tirkes T, Shah ZK, Takahashi N, et al. Reporting standards for chronic pancreatitis by using CT, MRI, and MR cholangiopancreatography: the consortium for the study of chronic pancreatitis, diabetes, and pancreatic cancer. Radiology 2019;290:207–215.
12. Vanga RR, Tansel A, Sidiq S, et al. Diagnostic performance of measurement of fecal Elastase-1 in detection of exocrine pancreatic insufficiency: systematic review and meta-analysis. Clin Gastroenterol Hepatol 2018; 16:1220–1228.e4.
13. 2. Classification and diagnosis of diabetes: standards of medical care in Diabetes-2022. Diabetes Care 2022; 45:S17–S38.
14. Hart PA, Yadav D, Li L, et al. High prevalence of osteopathy in chronic pancreatitis: a cross-sectional analysis from the PROCEED study. Clin Gastroenterol Hepatol 2022;20:2005–2013.
15. Hays RD, Bjorner JB, Revicki DA, et al. Development of physical and mental health summary scores from the patient-reported outcomes measurement information system (PROMIS) global items. Qual Life Res 2009;18:873–880.
16. Yadav D, Askew RL, Palermo T, et al. Association of chronic pancreatitis pain features with physical, mental, and social health. Clin Gastroenterol Hepatol 2023; 21:1781–1791.e4.
17. Ramsey ML, Lu A, Gumpper-Fedus K, et al. Clinical risk factors for sarcopenia in acute and chronic pancreatitis. BMC Gastroenterol 2025;25:60.
18. Marra-Lopez Valenciano C, Bolado Concejo F, Marín Serrano E, et al. Prevalence of exocrine pancreatic insufficiency in patients with chronic pancreatitis without follow-up. PANCR-EVOL study. Gastroenterol Hepatol 2018;41:77–86.
19. Erchinger F, Engjom T, Dimcevski G, et al. Exocrine pancreas insufficiency in chronic pancreatitis - risk factors and associations with complications. A multicentre study of 1869 patients. Pancreatology 2022;22:374–380.
20. Othman M, Kort J, Yu J, et al. Development of a clinical screening tool for exocrine pancreatic insufficiency in patients with chronic pancreatitis. Am J Gastroenterol 2022;117:S9.
21. Shetty R, Kumbhar G, Thomas A, et al. How are imaging findings associated with exocrine insufficiency in idiopathic chronic pancreatitis? Indian J Radiol Imaging 2022;32:182–190.
22. Dutta SK, Bustin MP, Russell RM, et al. Deficiency of fat-soluble vitamins in treated patients with pancreatic insufficiency. Ann Intern Med 1982;97:549–552.
23. Sikkens EC, Cahen DL, Koch AD, et al. The prevalence of fat-soluble vitamin deficiencies and a decreased bone mass in patients with chronic pancreatitis. Pancreatology 2013;13:238–242.
24. Martinez-Moneo E, Stigliano S, Hedstrom A, et al. Deficiency of fat-soluble vitamins in chronic pancreatitis: a systematic review and meta-analysis. Pancreatology 2016;16:988–994.
25. Ramai D, Facciorusso A, Maida M, et al. Prevalence of osteopathy in chronic pancreatitis: a systematic review and meta-analysis. Clin Transl Gastroenterol 2023;14: e00623.

Received April 11, 2026. Accepted September 18, 2026.

Correspondence:
Address correspondence to: Darwin L. Conwell, MD, MS, Division of Gastroenterology, Hepatology, and Nutrition, University of Kentucky, 900 S Limestone, Room 301, Lexington, Kentucky 40536. e-mail: Darwin.Conwell@uky.edu.

Acknowledgments:
The authors would like to acknowledge the continuous support by Dr Aynur Unalp-Arida as the program official for the Chronic Pancreatitis, Diabetes, and Pancreatic Cancer since its inception, as well as the past support of project scientist Dr Jose Serrano (2015–2025) and project consultant Dr Dana Andersen (2015–2025).

Authors' Contributions:
Mitchell L. Ramsey: Conceptualization, formal analysis, and writing – original draft preparation. Peng Yang: Data curation, formal analysis, investigation, methodology, and writing – original draft preparation. Liang Li: Data curation, formal analysis, investigation, and writing – review and editing. Dhiraj Yadav, Melena Bellin, William E. Fisher, Evan L. Fogel, Christopher E. Forsmark, Phil A. Hart, Walter G. Park, Stephen J. Pandol, Temel Tirkes, Mark Topazian, Stephen Van Den Eeden, and Santhi Swaroop Vege: Conceptualization, data

curation, investigation, and writing – review and editing. Radmila Choate and Rachel L. Hill: Data curation, investigation, and writing – review and editing. Darwin L. Conwell: Conceptualization, funding acquizing, resources, supervision, and writing – original draft preparation.

Conflicts of Interest:
The author discloses the following: Walter G. Park has consulted for Nestle Health Sciences, Amgen, Arctx Medical, Capsovision, mBIOTA, and Olympus.

Funding:
This work was supported by the National Cancer Institute (NCI) and National Institute of Diabetes and Digestive and Kidney Diseases (NIDDK) under award numbers: U01DK108288, U01DK108300, U01DK108306, U01DK108314, U01DK108323, U01DK108326, U01DK108327, U01DK108332, U01DK108320, U01DK108328, and U01DK144496. The content is solely the responsibility of the authors and does not necessarily represent the official views of the National Institutes of Health.

Ethical Statement:
The central institutional review board at MD Anderson as well as the institutional review board at each clinical center reviewed and approved the study (PROCEED study (NCT03099850)).

Data Transparency Statement:
Data may be made available upon reasonable request.

Reporting Guidelines:
STROBE.

Gastro Hep Advances, Volume *5*

Supplemental information

Exocrine Pancreatic Dysfunction in Chronic Pancreatitis: A Cross-Sectional Analysis of the Prospective Evaluation of Chronic Pancreatitis for Epidemiologic and Translational Studies Study (PROCEED)

Mitchell L. Ramsey, Dhiraj Yadav, Peng Yang, Liang Li, Melena Bellin, William E. Fisher, Evan L. Fogel, Christopher E. Forsmark, Phil A. Hart, Walter G. Park, Stephen J. Pandol, Temel Tirkes, Mark Topazian, Stephen Van Den Eeden, Santhi Swaroop Vege, Radmila Choate, Rachel L. Hill, and Darwin L. Conwell, on Behalf of the Consortium for the Study of Chronic Pancreatitis, Diabetes, and Pancreatic Cancer (CPDPC)

Title: Exocrine Pancreatic Dysfunction in Chronic Pancreatitis: Analysis of the PROCEED Study

SUPPLEMENTARY CONTENT

Supplementary Table 1: Baseline characteristics of CP participants with and without EPD in whom EPD status was assessed prior to or at enrollment, separated according to FE-1 result (<100, 100-200, and ≥200mcg/g) with overall 3-group comparison (Assessed Cohort, n=419).

	**FE-1 <100 (n=260)**	**FE-1 100-200 (n=41)**	**FE-1 ≥200 (n=118)**	**P-value (100-200 vs ≥200)**	**P-value (overall, 3-group)**
**Demographics**					
Median age at enrollment, y; median (IQR)	56 (46-63)	55 (47-61)	57 (45-65)	0.48	0.74
Female sex, n (%)	114 (43.8)	20 (48.8)	63 (53.4)	0.72	0.22
White race, n (%)	215 (82.7)	39 (95.1)	101 (85.6)	0.16	0.1
BMI, kg/m2; median (IQR)	23.9 (21.0-28.0)	25.4 (21.6-28.3)	25.2 (21.8-29.3)	0.75	0.09
**Social Determinants of Health**					
Highest level of education, n (%)				0.36	0.46
HS/GED or less	90 (34.9)	13 (32.5)	34 (29.3)		
Some training past HS	88 (34.1)	16 (40.0)	36 (31.0)		
Bachelor's or graduate school	80 (31.0)	11 (27.5)	46 (39.7)		
Family income, n (%)				0.91	0.02
Less than 50k	119 (49.8)	11 (31.4)	39 (35.5)		
50-100k	67 (28.0)	11 (31.4)	31 (28.2)		
100k or greater	53 (22.2)	13 (37.1)	40 (36.4)		
Marital status, n (%)				0.29	0.26
Single	59 (22.8)	8 (19.5)	24 (20.3)		
Married	140 (54.1)	29 (70.7)	70 (59.3)		
Divorced/Separated/Widowed	60 (23.2)	4 (9.8)	24 (20.3)		
Employment status, n (%)				0.51	**0.04**
Unemployed	97 (37.9)	12 (30.8)	25 (21.6)		
Employed	95 (37.1)	17 (43.6)	55 (47.4)		
Retired	64 (25.0)	10 (25.6)	36 (31.0)		
Disability or medical leave, n (%)	89 (35.6)	10 (27.0)	23 (22.3)	0.65	**0.04**
**Clinical Characteristics**					
Smoking status, n (%)				0.46	0.07
Current	98 (37.8)	11 (26.8)	30 (25.6)		
Past	78 (30.1)	16 (39.0)	35 (29.9)		
Never	83 (32.0)	14 (34.1)	52 (44.4)		
Charlson Comorbidity Index category, n (%)				0.35	**< .01**
1	159 (61.2)	37 (90.2)	95 (80.5)		
2	79 (30.4)	2 (4.9)	16 (13.6)		
3	22 (8.5)	2 (4.9)	7 (5.9)		
Diabetes, n (%)	138 (55.0)	12 (33.3)	29 (26.1)	0.4	**< .01**
PERT usage, n (%)	184 (71.0)	16 (40.0)	29 (25.0)	0.1	**< .01**
Prior AP, n (%)	206 (82.7)	34 (85.0)	100 (85.5)	1	0.81
Duration of AP prior to enrollment, y; median (IQR)	5 (2-11)	3 (1-10)	3 (1-11)	0.95	0.06

Etiology, n (%)				0.12	**< .01**
Alcohol	126 (48.5)	14 (34.1)	25 (21.2)		
Idiopathic	92 (35.4)	14 (34.1)	61 (51.7)		
All others combined	42 (16.2)	13 (31.7)	32 (27.1)		
Pancreatic calcifications, n (%)	221 (85.0)	23 (56.1)	70 (59.3)	0.72	**< .01**
Pancreas size on imaging, n (%)				**< .01**	**< .01**
<7mm	52 (20.0)	3 (7.3)	7 (6.0)		
7-14mm	117 (45.0)	22 (53.7)	28 (23.9)		
>14mm	91 (35.0)	16 (39.0)	82 (70.1)		
Endoscopic therapy, n (%)	145 (56.4)	24 (60.0)	47 (40.5)	**0.04**	**0.01**
Pancreatic surgery, n (%)	35 (13.5)	4 (9.8)	4 (3.4)	0.21	**< .01**

Supplemental Table 2: Comparison of chronic pancreatitis-related features and outcomes between subjects with and without exocrine pancreatic dysfunction (EPD) diagnosed according to clinical features or study fecal elastase (FE-1) testing (Assessed Cohort). *Data presented as n (%) unless otherwise noted.*

	**FE-1 < 100 mcg/g (n = 260)**	**FE-1 ≥ 100 mcg/g (n = 159)**	***P*-value**	**FE-1 < 200 mcg/g (n = 301)**	**FE-1 ≥ 200 mcg/g (n = 118)**	***P*-value**
**Metabolic Bone Disease**						
Low vitamin D, *n (%)*	61 (39.9)[104]	24 (33.8)[87]	.46	69 (40.1)[129]	24 (33.8)[88]	.39
DXA result, *n (%)*						
Normal	67 (42.1)[101]	38 (47.5)[79]		78 (43.8)[123]	27 (44.3)[57]	
Osteopenia	59 (37.1)	33 (41.3)		64 (36.0)	28 (25.9)	
Osteoporosis	33 (20.8)	9 (11.2)	.19	36 (20.2)	6 (9.8)	.14
DXA result, *n (%)*						
Normal	67 (42.1)[101]	38 (47.5)[79]		78 (43.8)[123]	27 (44.3)[57]	
Abnormal	92 (57.9)	42 (52.5)	.81	100 (56.2)	34 (55.7)	1.00
Spontaneous fracture, *n (%)*	4 (1.5)[1]	4 (2.5)	.49	4 (1.3)[1]	4 (3.4)	.23
Traumatic fracture, *n (%)*						
Yes	81 (31.5)[3]	54 (34.2)[1]		96 (32.2)[3]	39 (33.3)[1]	
No	176 (68.5)	104 (65.8)	.59	202 (67.8)	78 (66.7)	.81
Traumatic fracture^ *n (%)*						
Minor	34 (33.0)[7]	27 (42.9)[1]		42 (35.3)[7]	19 (40.4)[1]	
Moderate	45 (43.7)	26 (41.3)		50 (42.0)	21 (44.7)	
Major	24 (23.3)	10 (15.9)	.35	27 (22.7)	7 (14.9)	.54
Traumatic fracture with osteopathy, *n (%)*						
Minor	15 (39.5)[3]	11 (44.0)[1]		17 (39.5)[3]	9 (45.0)[1]	
Moderate	15 (39.5)	12 (48.0)		18 (41.9)	9 (45.0)	
Major	8 (21.0)	2 (8.0)	.45	8 (18.6)	2 (10.0)	.77

^among 135 subjects with any traumatic fracture.

Note: Categorical variables are summarized as count (percentage of available data).

Note: The number of subjects with missing data is annotated in superscript.

Note: P-value is from comparison between EPD and no-EPD group, with the Fisher exact test for categorical variables.

Note: Abnormal Osteopathy includes subjects with any osteopenia or osteoporosis.

Supplementary Figure 1. Receiver operator characteristic (ROC) curve for EPD diagnosis (FE-1 ≤100mcg/g) based on the multivariate logistic regression from Table 3, A) with PERT use variable, and B) without PERT use variable.

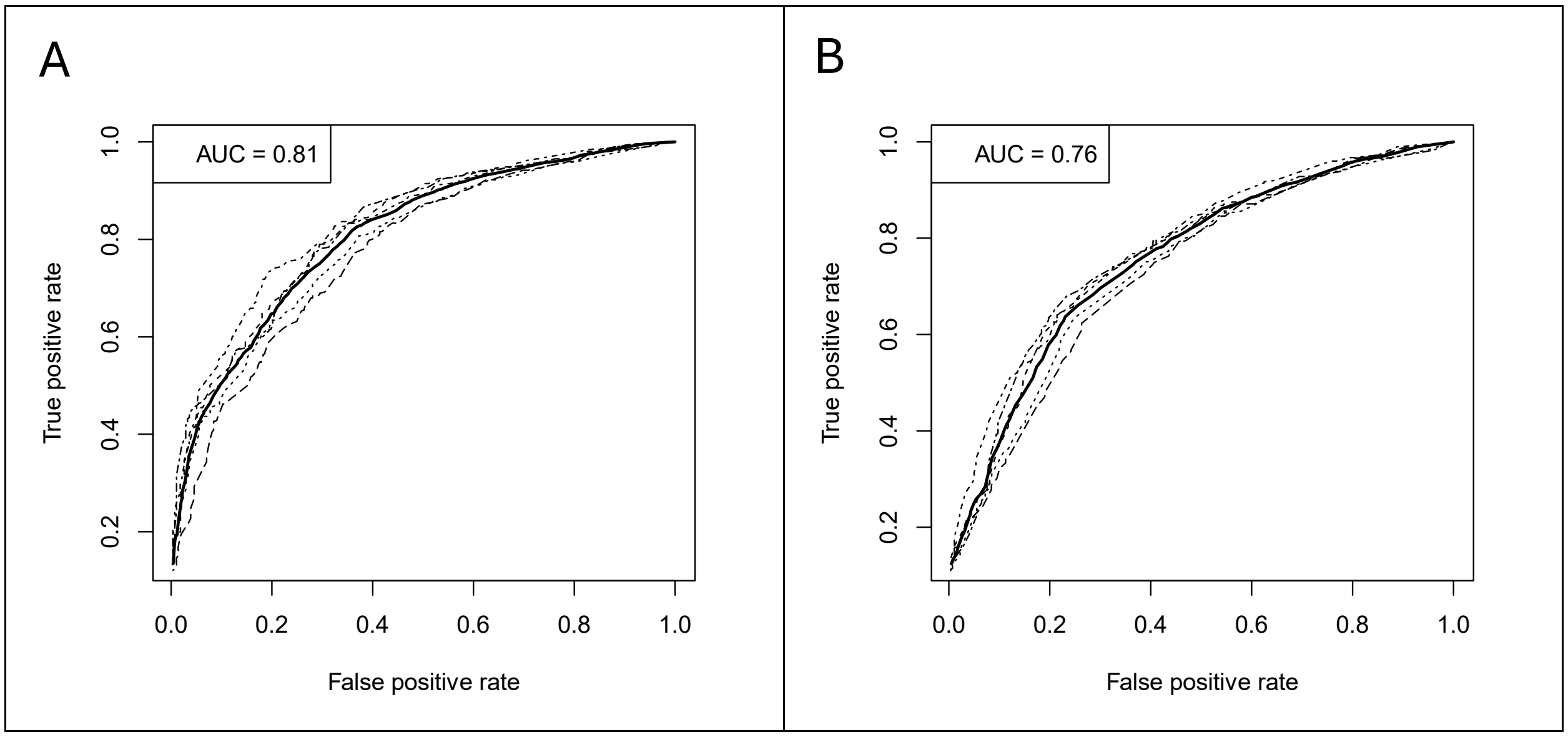

Supplement: Supplementary file 2 [file mmc2.pdf]
